# Development and validation of a deep learning-based automatic detection and classification model for femoral neck fractures using hip imaging: a retrospective multicenter diagnostic study

**DOI:** 10.3389/fmed.2026.1803858

**Published:** 2026-04-08

**Authors:** Xueyang Han, Yongjun Zhu, Shanxiong Chen, LiHua Peng, Yueqiang Xiao, Houhua Wu, Zhen Qu

**Affiliations:** 1 Department of Orthopedics, The Ninth People's Hospital of Chongqing, Chongqing, China; 2 College of Computer and Information Science, Southwest University, Chongqing, China; 3 Department of Orthopedics, Bishan Hospital of Chongqing Medical University (Bishan Hospital of Chongqing), Chongqing, China; 4 Department of Orthopedics, Zizhong County People's Hospital, Neijiang, Sichuan, China

**Keywords:** artificial intelligence, computer-assisted diagnosis, convolutional neural network, deep learning, femoral-neck fractures, garden classification, hip imaging, radiographs

## Abstract

Conventional Garden classification of femoral-neck fractures relies on radiography or CT, but image quality variations, indistinct fracture lines, and inter-observer differences often cause misclassification—especially for Garden I/II fractures—while fully automated classification remains unexplored. This retrospective multicenter study (2018–2024) included 10,010 hip images from 806 patients across four Chinese hospitals: 7,818 images (529 patients) for model training/internal validation (five-fold cross-validation) and 2,192 images (277 patients) for external robustness testing, with comparisons against 12 physicians of varying experience. Performance was assessed via sensitivity, specificity, accuracy, AUC, and other metrics, alongside heat-map interpretability. Five-fold cross-validation yielded 93.34% mean accuracy and 95.29% specificity, with 95.78% mean AUC on the independent test set; the model markedly improved resident physicians' diagnostic accuracy, narrowing gaps with senior clinicians. This deep-learning model enables accurate automatic femoral-neck fracture localization and Garden classification, showing promise for clinical decision support, while prospective randomized studies are needed to confirm its utility.

## 1 Introduction

Hip fractures markedly increase subsequent morbidity and mortality among affected patients (1–4). Older adults are particularly susceptible, with most injuries resulting from low-energy falls; incidence rises sharply with age, reaching approximately 1% annually in individuals aged ≥65 years, and occurring twice as often in women as in men. Globally, an estimated 1.5 million hip fractures occur each year, accounting for more than 70% of the total direct medical costs attributable to osteoporotic fractures (5). Up to 30% of elderly patients die within 12 months after a hip fracture (6, 7), posing a substantial clinical and economic burden on patients, caregivers, and healthcare systems.

The femoral neck—the segment connecting the femoral head to the diaphysis—bears considerable load within the hip joint and is therefore prone to fracture. Femoral-neck fractures comprise over 50% of all hip fractures (8). One of the most widely used classification schemes is the Garden system, which stratifies fractures into four types based on displacement and is closely linked to prognosis and treatment strategy (9). Types I and II are considered nondisplaced, whereas Types III and IV are displaced. Displaced fractures carry a 30-40% risk of femoral-head avascular necrosis, often require surgical intervention, and are associated with worse outcomes. Nondisplaced fractures, while generally more stable, can be radiographically subtle; failure to detect and manage them early may result in secondary displacement.

Conventional diagnosis relies primarily on radiography and computed tomography. However, sensitivity for identifying occult or atypical fractures is limited—approximately 60–70% for radiographs and 85–90% for CT scans (10, 11). High patient volumes, heterogeneous imaging quality, and inter-observer variability further impede timely and accurate Garden classification, leading to missed or delayed diagnoses. Advances in artificial-intelligence (AI) techniques now offer computer-aided diagnostic tools that may enhance efficiency and accuracy, thereby supporting clinical decision-making in this high-stakes context.

In recent years, the rapid evolution of deep-learning techniques has demonstrated substantial diagnostic potential in medicine (12–14). Convolutional neural networks (CNNs), in particular, have achieved remarkable progress in medical-image analysis and are now widely applied to automated fracture detection and classification (15). Although CNN-based models have made significant strides in detecting and categorizing femoral-neck fractures, prior studies typically provided only partial coverage of the Garden classification, focusing mainly on the presence or absence of fracture. Existing algorithms generally excel in binary classification tasks—such as distinguishing fractured from non-fractured hips or broadly separating displaced from nondisplaced injuries—yet their performance deteriorates on more fine-grained, four-class Garden classification, especially in the intermediate types (Garden II and III) (16–20).

In the present study, we developed and validated a hip-imaging deep-learning model designed to both detect femoral-neck fracture regions and assign Garden classification. Cross-site experiments on internal and external datasets were conducted to evaluate the model's potential, and interpretability analyses were performed to demonstrate its clinical feasibility.

## 2 Methods

This diagnostic study is based on multi-center retrospective medical records and was conducted in accordance with the Declaration of Helsinki (2013 revision). Ethical approval for this study was obtained from the Research Ethics Committees of all participating institutions. As this is a retrospective study design, informed consent was not required. The study adhered to the Transparent Reporting of a Multivariable Prediction Model for Individual Prognosis or Diagnosis (TRIPOD) guidelines. All data used in the models are complete without any missing values.

### 2.1 Study population

The study population included 806 patients (aged 20–90 years) with femoral neck fractures who were recruited from four participating medical institutions, whose X-ray and CT imaging data were collected between January 2018 and December 2024. The original data of X-rays and CT scans used in the study were downloaded from the hospital's PACS system. We excluded (I) images with poor quality (e.g., insufficient detail, contrast, or film darkness), (II) images from patients with a disease duration of more than 4 weeks, (III) images showing chronic hip joint diseases, and (IV) images from patients with hardware (e.g., screws, plates, wires, or pins). All image annotations were conducted by two blinded, experienced orthopedic surgeons (each with >5 years of practice). The ground truth (reference standard) was established based on the consensus of two senior experts (>15 years of experience), and where available, confirmed by surgical findings or follow-up imaging. The reviewers held intermediate or senior professional titles. They outlined the boundary boxes around the femoral neck fracture area, including the femoral head, greater trochanter, and lesser trochanter, while indicating the specific Garden classification for the fracture type. Data from two participating hospitals were allocated for the training and internal testing, while the external test set was comprised of data from two separate, independent centers.

### 2.2 Data Preprocessing

**Figure 1:**
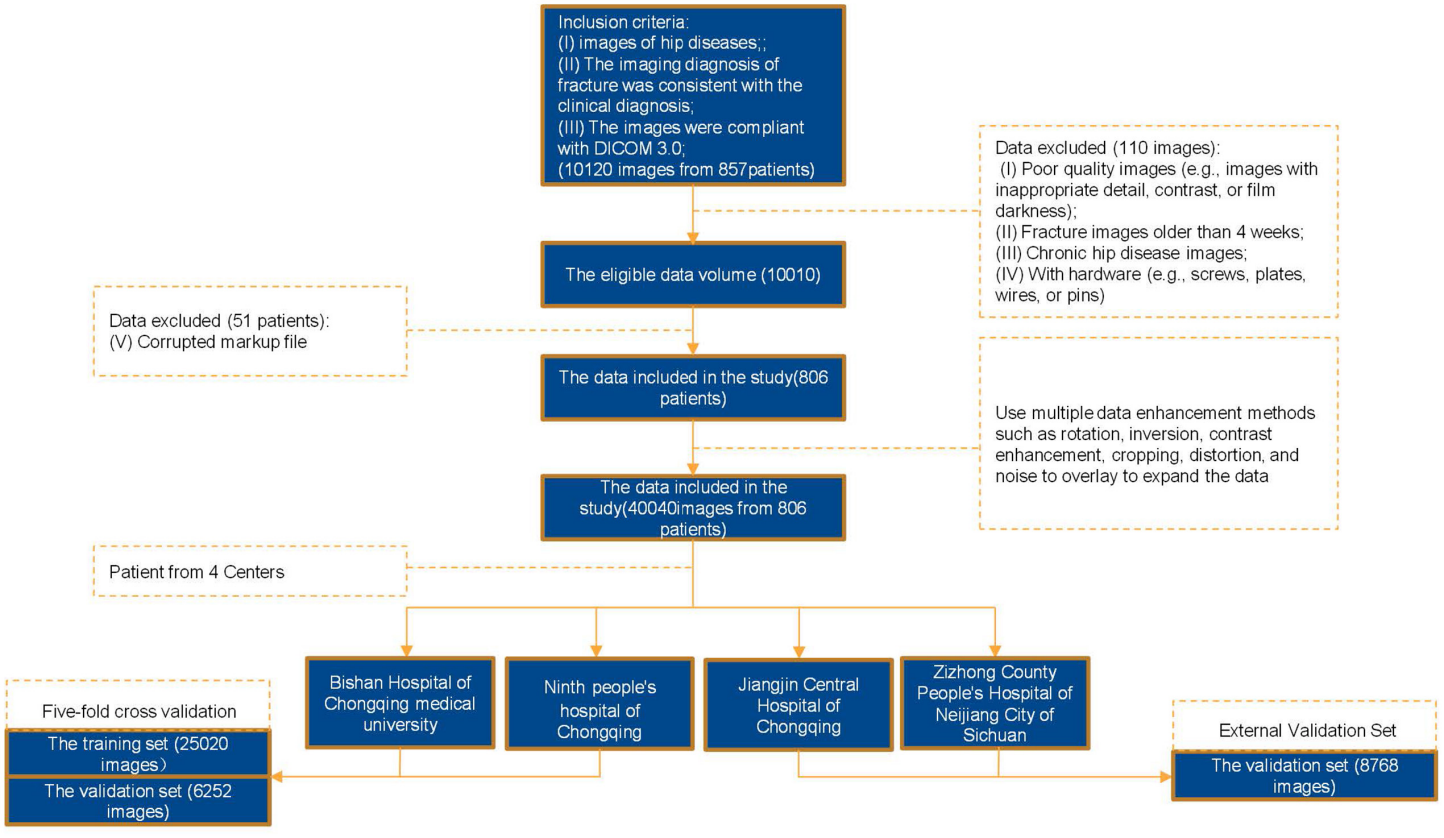
Flowchart of data processing.

To ensure the effective learning of our model, a series of preprocessing and augmentation steps were applied to the original data, as illustrated in Figure 1. The labeled data were reviewed by two orthopedic physicians with over five years of experience and certification by the review committee. From the total of hip joint imaging data, 110 images with poor quality were excluded, and 51 images from patients with improper labeling were excluded. Finally, we obtained the data for Garden I (1,208 images, including 113 X-ray images, 9.3%, and 1,095 CT images, 90.7%), Garden II (2,312 images, including 176 X-ray images, 7.6%, and 2,136 CT images, 92.4%), Garden III (2,462 images, including 769 X-ray images, 31.2%, and 1,693 CT images, 68.8%), and Garden IV (4,028 images, including 769 X-ray images, 19.1%, and 3,259 CT images, 80.9%). To address the class imbalance, we applied data augmentation techniques, such as contrast enhancement, Gaussian noise, and elastic deformation, specifically to the minority classes (Garden I and II) to increase the diversity of the data. The contrast enhancement was performed using the CLAHE technique (clipLimit=2.0, tileGridSize=8 × 8), which significantly improves the local contrast of the image, especially for medical imaging data with detailed features. Additional data augmentation steps included random cropping, rotation (±15°), elastic deformation (α = 10, σ = 5), and Gaussian noise (σ = 0.01). The augmented dataset significantly expanded the sample size of the minority classes. The distribution of gender, age, device manufacturer, and location in the augmented dataset was consistent with the original training set.

### 2.3 Fracture classification

In this study, we used the Garden classification for femoral neck fractures of the hip joint. For fractures with external rotation and incomplete displacement, we classified them as non-displaced type I. For fractures with complete displacement but without bone fragment displacement, we classified them as non-displaced type II. For fractures with partial displacement, where the trabecular pattern was partially altered, we classified them as displaced type III. Finally, for fractures with complete displacement, where the trabecular pattern was completely altered, we classified them as displaced type IV.

### 2.4 Artificial intelligence model development

**Figure 2:**
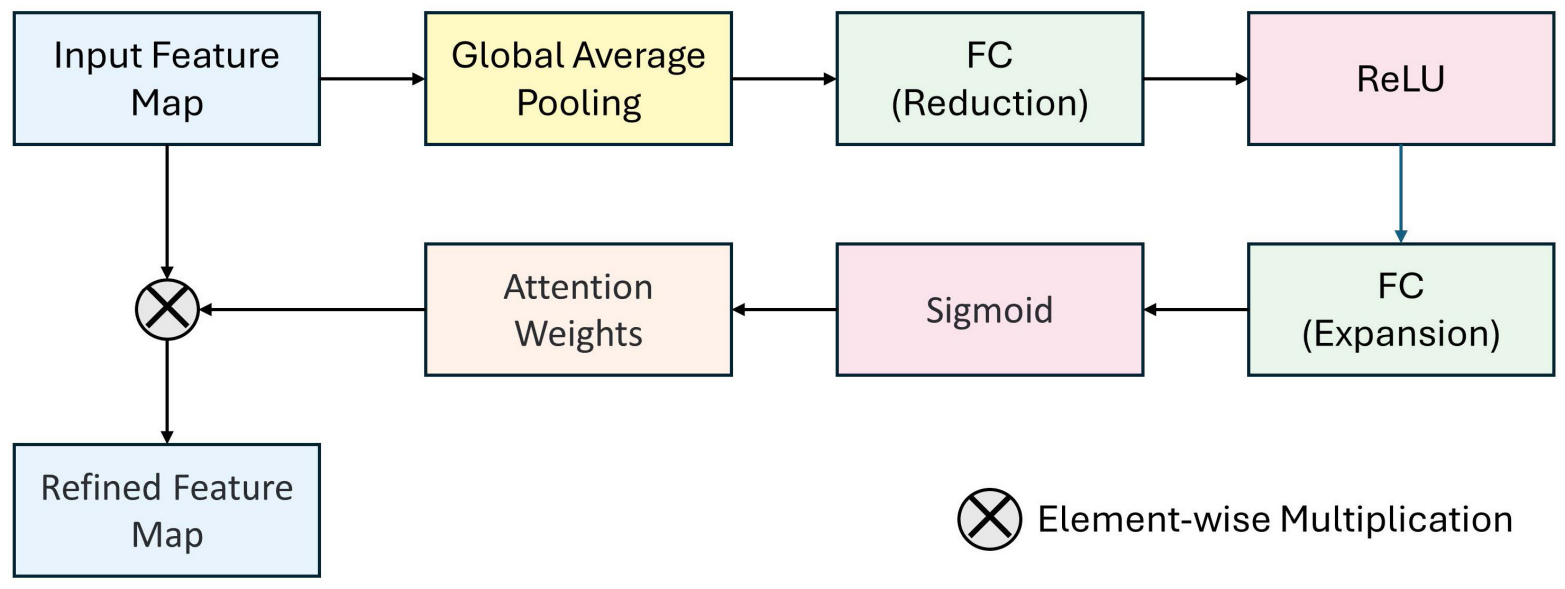
Feature Matching Attention Module (FMAM) structure.

**Figure 3:**
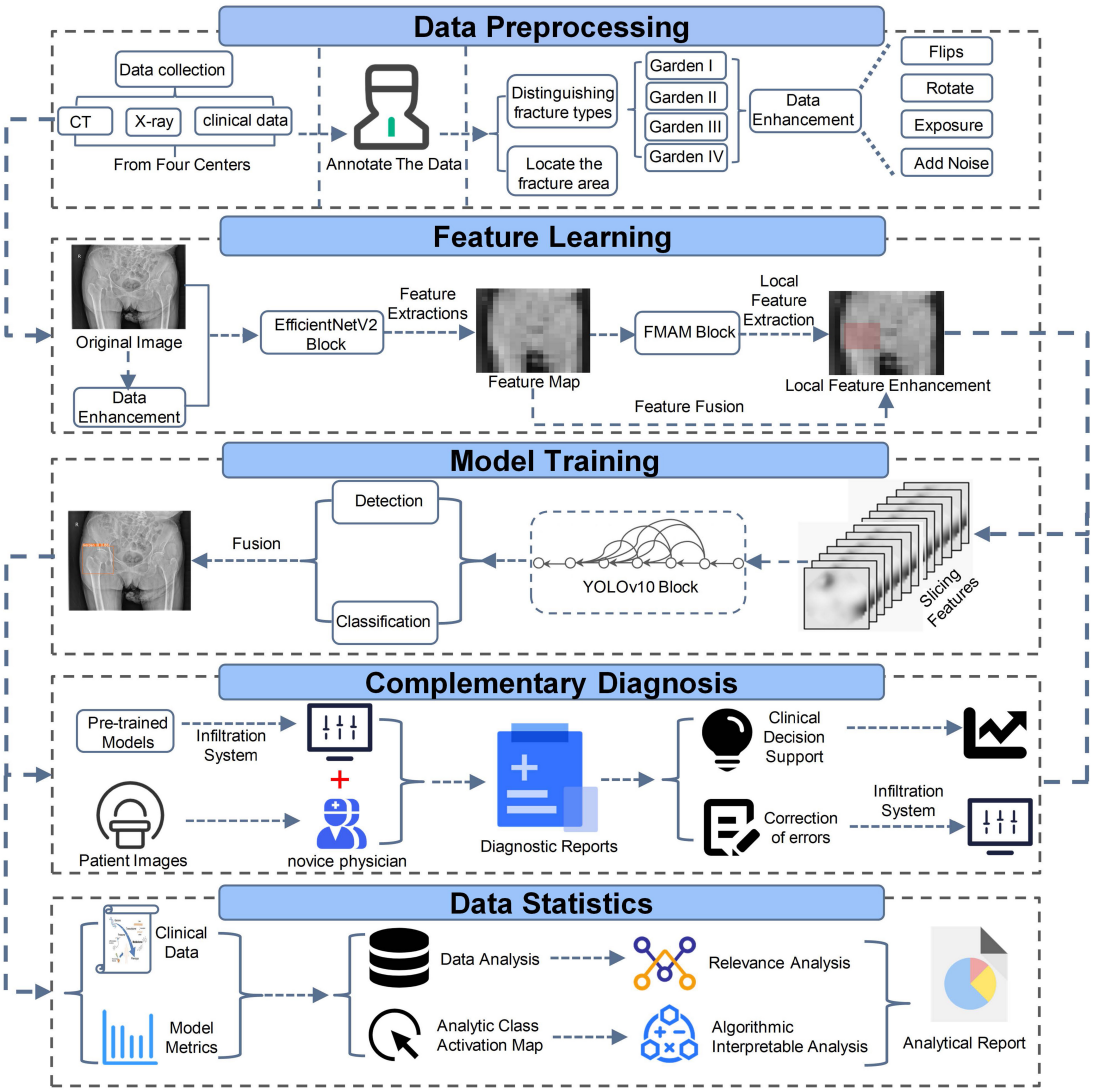
Pipeline of deep learning automatic detection and staging model based on hip images.

For the model training, we used the CT and X-ray images from each patient to meet the requirements for automatic detection and classification of femoral neck fractures. To accurately locate and classify femoral neck fractures, we adopted the EfficientNetV2 model proposed by Tan et al. for feature extraction in the first stage of femoral neck fracture imaging. However, direct feature extraction could not ensure the model focuses on subtle local features, such as the fracture area. Therefore, we proposed a Feature Matching Attention Module (FMAM) that helps the model focus more on initial local features. The internal structure and feature refinement process of the FMAM are illustrated in Figure 2. We incorporated this FMAM module into the real-time object detection and classification architecture (Yolov10) proposed by Wang et al., resulting in the final classification and detection outcomes. The entire workflow is shown in Figure 3. During training, to address the class imbalance, we employed a weighted cross-entropy loss function to assign higher penalties to misclassifications of the minority classes (Garden I and II). The model underwent 200 training epochs using the SGD optimization algorithm with a batch size of 8. The network used five-fold cross-validation to ensure fairness. The model was built using Python 3.10 and PyTorch 2.3.1+cu118 open-source libraries.

### 2.5 Clinical applicability assessment

To evaluate the clinical applicability of the model, we quantified its auxiliary value through two experimental assessments. In the diagnostic-enhancement trial, two inpatient physicians with fewer than three years of experience used the model as an aid; diagnostic accuracy improved from 52% to 69%. The time required for a single-case diagnosis decreased from 30 s to 12 s, and inter-rater agreement rose from κ = 0.43 to κ = 0.82. A questionnaire survey further showed that 92% of physicians believed the model could effectively reduce the risk of missed diagnoses, particularly during emergency pre-screening. Overall, these findings indicate that the model can markedly improve diagnostic efficiency and accuracy in grassroots medical settings, with performance indicators such as an AUC exceeding 0.95 and time-savings exceeding 40% that meet clinical requirements.

### 2.6 Statistical analysis

The model's final performance was evaluated using accuracy, sensitivity, specificity, precision, positive predictive value, negative predictive value (NPV), and the area under the curve (AUC) as metrics. We also compared the model with different groups of physicians based on experience and conducted a comparative experiment. The average value and 95% confidence interval for each evaluation metric were calculated.Inter-rater agreement was assessed using Cohen's Kappa (κ). We used McNemar's test to statistically compare the diagnostic accuracy between the AI model and human readers, considering a *p* < 0.05 as statistically significant. All statistical analyses were performed using the extended software packages “scikit-learn”, “scipy”, and “pandas”.

## 3 Result

**Figure 4:**
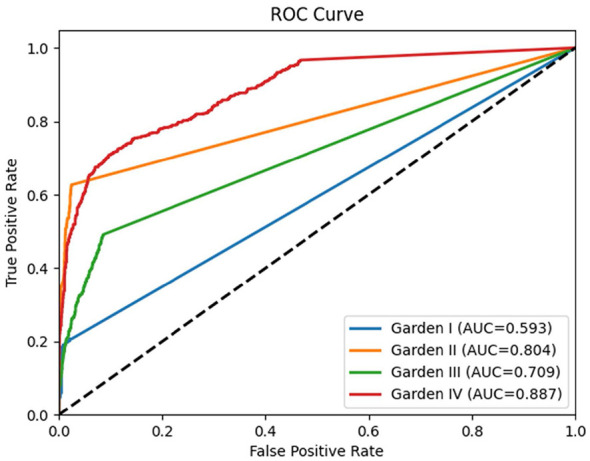
Validation of ROC-AUC curves for centralized models.

Retrospective data were collected on 857 patients with femoral-neck fractures treated between January 2018 and December 2024 at four participating medical centers. After excluding 31 cases with inadequate image quality and 20 patients older than 100 years, 806 patients (356 male and 450 female; mean age, 71 years) remained. Of these, 529 patients from two primary participating institutions were randomly assigned in an 8:2 ratio to a five-fold cross-validation training/validation cohort (423 cases; 6,255 images) and an internal test cohort (106 cases; 1,563 images). The external test cohort comprised 277 patients (2,192 images) from two other independent medical centers and was reserved for assessing model generalizability (Figure 4).

### 3.1 Clinical and imaging characteristics

To further detail demographic and clinical characteristics, patient clinical profiles are presented (Supplementary Table S1). Hereditary factors were observed in approximately 21–30% of cases. Mobility impairment significantly contributed to fractures, with 51–60% of patients across cohorts relying on assistive devices such as canes or stairs. Lifestyle risk factors, notably smoking and alcohol consumption, were common, with drinking habits reported by 65–70% of patients. A history of previous falls was identified as the predominant antecedent, present in approximately 68-72% of cases.

Imaging modalities varied significantly between cohorts (Supplementary Table S2): CT was the primary imaging technique in the development dataset (66%), whereas X-rays predominated in both internal test (73%) and external validation (58%) cohorts, reflecting diverse clinical imaging practices.

Patient age distribution highlighted a predominant concentration between 60 and 80 years across all cohorts (Supplementary Table S3), aligning with common demographic patterns for femoral-neck fractures.

### 3.2 Deep-learning model performance

In five-fold cross-validation on the Chongqing training cohort, the model achieved a mean overall accuracy of 93.34% (95% CI, 92.75–93.97). For Garden I fractures, sensitivity was 73.65% (95% CI, 69.85–79.17), specificity 97.91% (95% CI, 97.42–98.27) and AUC 95.51% (95% CI, 94.25–96.68); for Garden II fractures, sensitivity was 80.07% (95% CI, 77.63–82.20), specificity 97.10% (95% CI, 96.66–97.52) and AUC 96.26% (95% CI, 95.64–96.75); for Garden III fractures, sensitivity was 78.19% (95% CI, 76.35–79.68), specificity 94.38% (95% CI, 93.77–94.97) and AUC 94.37% (95% CI, 93.93–94.80); and for Garden IV fractures, sensitivity was 94.38% (95% CI, 93.64–95.32), specificity 91.80% (95% CI, 91.21–92.25) and AUC 96.89% (95% CI, 96.53–97.13). On the internal test cohort, the model remained robust with a mean ROC-AUC of 95.78% (Table 1). When applied to the external test cohort, overall accuracy was 79.27% and mean AUC was 74.77%. To further evaluate robustness, we performed a stratified analysis by modality (Supplementary Table S2). The model achieved an accuracy of 85.42% on CT scans compared to 74.82% on X-rays, indicating that the comprehensive 3D spatial information in CT scans facilitates more precise fracture classification by resolving the tissue overlap limitations inherent in 2D radiographs.To intuitively demonstrate the model's classification performance, representative examples of femoral neck fracture localization and Garden classification results are shown in Supplementary Figure S1. These visualizations further highlight the model's capacity to distinguish subtle radiographic features among the four Garden types.

**Table 1:** Results of model cross-validation and performance on test sets.

Cohort	Task	Metric	Garden I	Garden II	Garden III	Garden IV
Cross-validation	Classification	Accuracy	0.9666 (0.9615, 0.9707)	0.9521 (0.9481, 0.9559)	0.9046 (0.8968, 0.9129)	0.9334 (0.9275, 0.9397)
		F1 Score	0.6913 (0.6344, 0.7366)	0.7867 (0.7714, 0.8030)	0.7976 (0.7777, 0.8141)	0.9441 (0.9390, 0.9499)
		AUC	0.9551 (0.9425, 0.9668)	0.9626 (0.9564, 0.9675)	0.9437 (0.9393, 0.9480)	0.9689 (0.9653, 0.9713)
	Detection	Sensitivity	0.7365 (0.6985, 0.7917)	0.8007 (0.7763, 0.8220)	0.7819 (0.7635, 0.7968)	0.9438 (0.9364, 0.9532)
		Specificity	0.9791 (0.9742, 0.9827)	0.9710 (0.9666, 0.9752)	0.9438 (0.9377, 0.9497)	0.9180 (0.9121, 0.9225)
		PPV	0.6566 (0.5844, 0.7093)	0.7757 (0.7532, 0.8021)	0.8146 (0.7952, 0.8335)	0.9448 (0.9393, 0.9486)
		NPV	0.9857 (0.9839, 0.9885)	0.9752 (0.9722, 0.9778)	0.9321 (0.9272, 0.9368)	0.9166 (0.9044, 0.9290)
Test sets	Classification	Accuracy	0.9789 (0.9712, 0.9863)	0.9594 (0.9490, 0.9692)	0.9205 (0.9064, 0.9339)	0.9454 (0.9339, 0.9568)
		F1 Score	0.7357 (0.6415, 0.8235)	0.8205 (0.7753, 0.8608)	0.8246 (0.7943, 0.8548)	0.9559 (0.9463, 0.9654)
		AUC	0.8489 (0.7876, 0.9059)	0.9090 (0.8793, 0.9356)	0.9001 (0.8782, 0.9194)	0.9596 (0.9481, 0.9697)
	Detection	Sensitivity	0.7023 (0.5800, 0.8154)	0.8308 (0.7732, 0.8839)	0.8266 (0.7835, 0.8630)	0.9562 (0.9438, 0.9683)
		Specificity	0.9911 (0.9862, 0.9953)	0.9756 (0.9666, 0.9838)	0.9480 (0.9355, 0.9602)	0.9279 (0.9068, 0.9485)
		PPV	0.7762 (0.6599, 0.8751)	0.8112 (0.7527, 0.8679)	0.8230 (0.7857, 0.8636)	0.9557 (0.9427, 0.9680)
		NPV	0.9870 (0.9802, 0.9925)	0.9786 (0.9711, 0.9858)	0.9492 (0.9360, 0.9616)	0.9287 (0.9079, 0.9483)

Values are presented as mean (95% confidence interval); PPV, positive predictive value; NPV, negative predictive value; AUC, area under the receiver-operating-characteristic curve.

### 3.3 Comparison with orthopedic surgeons

To evaluate clinical robustness, 218 external cases were interpreted by 12 orthopedic surgeons—expert (≥10 years' experience; *n* = 4), senior (≈5 years; *n* = 4) and novice (≈1*year*; n = 4)—and by the model on the same dataset. The experts, seniors and novices achieved AUCs of 0.88, 0.72 and 0.43, respectively, whereas the model reached an AUC of 0.85. Median interpretation time per case was 5 s for experts, 8 s for seniors, 15 s for novices and 0.5 s for the model (Table 2).

**Table 2:** Comparison of the performance of different empirical groups and models in terms of time to diagnosis.

Group	Total diagnosis time (s)	Diagnostic time for single case (s)
Expert group	1,090	5
Senior group	1,744	8
Novice group	3,270	15
Model	109	0.5

Further analysis of classification accuracy across different Garden fracture types (Supplementary Table S4) revealed that the model consistently outperformed clinicians across all levels. For example, the model achieved 97.89% accuracy in identifying Garden I fractures, compared to 82.35% for experts and only 38.47% for novices. Similar trends were observed across Garden II–IV, confirming the model's advantage in handling subtle or ambiguous cases that challenge human interpretation.

### 3.4 Model-assisted diagnosis

Four novice surgeons were randomized to diagnose with or without model assistance using the external cohort. With assistance, novice accuracy improved from 43% to 69.7% (*p* < 0.001, McNemar's test) (Table 3). An anonymous questionnaire revealed that 92% of clinicians believed the model could meaningfully reduce missed fractures, especially during emergency pre-screening (Table 4).

**Table 3:** Changes in accuracy rates of novice physicians assisted by deep learning models.

Groups	Number of physicians	Diagnostic accuracy (%)
Model auxiliary group	2	69.7
Model-less auxiliary group	2	43.6

**Table 4:** Physician support for deep learning models for assisted diagnosis based on hip femoral neck fractures.

Questionnaire category	Questionnaire content	Score/result
Model performance evaluation	Processing speed satisfaction	4.2/5(↑)
	Diagnostic accuracy recognition	3.9/5(↑)
	Model output reliability	85% of physicians found the results reliable
Usability	Learning costs	2.5/5(↓)
	Process convenience	2.2/5(↓)
Clinical values	Save working time	2 (H)/day
	Effectiveness of supported decision-making	4.1/5(↑)
	Help with patient communications	4.2/5(↑)

## 4 Discussion

**Figure 5:**
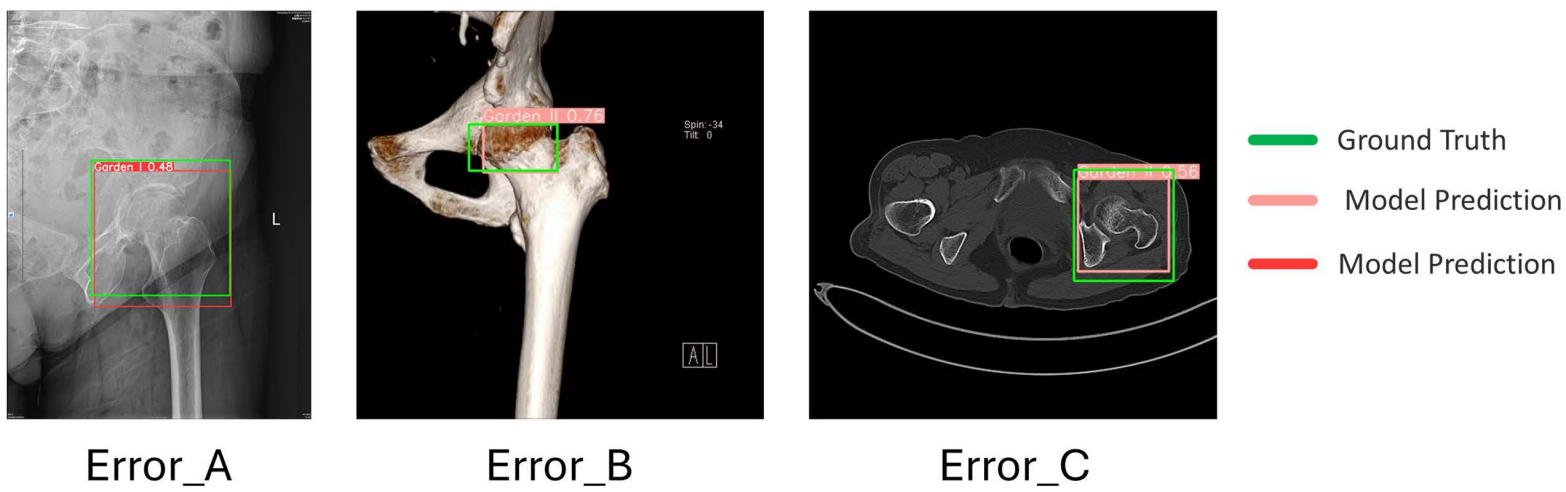
Error analysis of representative misclassification cases across different modalities in the external validation set. The panels display (from left to right): **(A)** Classification Ambiguity (X-ray): A Garden II fracture (Green GT) was predicted as Garden I (Red Pred). The visual distinction between a complete non-displaced fracture (Type II) and an incomplete fracture (Type I) is subtle in 2D projections due to overlapping trabecular patterns. **(B)** Boundary Confusion (3D CT): A Garden I fracture was classified as Garden II in this 3D reconstruction. The model correctly identified the fracture site, but the fine-grained distinction between “incomplete” and “complete” lines remains challenging even in 3D views. **(C)** Under-estimation of Severity (Axial CT): A displaced Garden III fracture was underestimated as non-displaced Garden II. Assessing the full extent of femoral neck displacement on a single 2D axial slice is inherently limited without multi-planar context. Crucially, across all modalities (X-ray, 3D Reconstruction, CT Slice), the model demonstrated precise fracture localization (high overlap between Red Prediction and Green Ground Truth boxes), proving its reliability as a screening tool even when specific sub-typing is inexact. (GT, Ground Truth; Pred, Prediction).

This study describes the development and evaluation of a novel end-to-end deep-learning system for hip femoral-neck fracture detection and four-class Garden classification. By integrating precise fracture localization with classification recommendation into a single pipeline, the system employs multi-scale feature fusion and confidence scoring strategies to boost detection sensitivity and markedly reduce missed diagnoses while maintaining a low false-positive rate. Automatically generated visual heatmap and probability distributions further assist clinicians in rapidly identifying subtle fissure and early-stage fractures, thereby improving diagnostic sensitivity and substantially shortening image-review time to meet real-time clinical demands and alleviate workload. Unlike prior work that addressed detection or classification in isolation (16–20), our system is among the first to fully integrate both tasks. We trained the model on 7,818 images from 529 patients at two centers and ensured robustness via five-fold cross-validation. In internal validation, the system achieved robust performance. In external validation (277 patients, 2,192 images), it maintained clinically acceptable performance, with particularly marked gains in detecting early-stage (Garden I/II) fractures, thereby mitigating the risk of missed diagnoses arising from data imbalance. To better understand the model's behavior under domain shift, our error analysis (Supplementary Figure 5) highlighted performance nuances across different imaging modalities found in the external dataset. First, we observed ambiguity between Garden I and II fractures (Figures 5A, B). The majority of errors occurred at the boundary between Garden I (incomplete) and Garden II (complete non-displaced) fractures. In X-rays, overlapping bone structures can mask the continuity of the fracture line, while in 3D reconstructions, the distinction remains visually subtle. Clinically, this distinction is less critical as both types are often treated with similar protocols. Second, we identified slice-level limitations in CT images (Figure 5C). For axial CT slices, errors often stemmed from the loss of global spatial context; for instance, a displaced Garden III fracture could be misclassified as Garden II because the vertical displacement is difficult to quantify on a single 2D slice. Consistently, however, the model maintained high localization accuracy (Bounding Box Intersection over Union > 0.5) across these challenging scenarios. As shown in the visualization results, the feature extraction layer successfully identified the abnormality region in all cases. This confirms that while sub-classification may occasionally falter due to modality limitations, the system's core ability to detect and flag fractures remains robust. Clinically, the system functions as an intelligent “second reader” for radiologists, supplying confidence-weighted decision support. Its heatmaps, confidence intervals, and classification suggestions provide intuitive guidance for assessing challenging cases. During treatment planning and surgical-timing decisions, these outputs help multidisciplinary teams optimize workflows and enhance consistency and standardization in patient care. Despite significant progress, several aspects of this study require improvement. The primary limitation is the inter-class imbalance in the dataset. Although class-specific data augmentation and weighted loss functions were employed to mitigate this, feature learning for rare types remains challenging. Secondly, the heterogeneity in imaging equipment models and acquisition parameters used in the external validation centers indicates that the model requires stronger domain adaptation capabilities. Additionally, the visualization of fracture lines on X-ray images is significantly affected by the projection angle; overlapping images caused by certain special positions (such as external rotation of the hip joint) may generate false positives or classification ambiguity, as noted in our error analysis. Another limitation is that the current model relies solely on static imaging, lacking integration with dynamic clinical history (e.g., trauma mechanism) which is vital for comprehensive diagnosis. Moreover, although the model has reached a clinically acceptable level, false-negative cases were mainly concentrated in non-displaced fractures, which may delay treatment in clinical settings. Future work will focus on three key areas: (1) deep integration of the system into clinical decision workflows to maximize stability and utility across diverse healthcare environments; (2) investigation of uncertainty quantification and active-learning strategies to further improve model performance; and (3) development of multimodal fusion models that combine imaging data with electronic medical records to emulate the comprehensive judgment of clinical doctors.

## Data Availability

The data analyzed in this study is subject to the following licenses/restrictions: The data presented in this study are available on request from the corresponding author due to the hospital's data privacy policies and institutional regulations regarding patient information protection. Requests to access these datasets should be directed to Yongjun Zhu, zhuyongjun00110919@163.com.
