# Correction: Development and validation of a deep learning-based automatic detection and classification model for femoral neck fractures using hip imaging: a retrospective multicenter diagnostic study

**DOI:** 10.3389/fmed.2026.1857321

**Published:** 2026-05-01

**Authors:** Xueyang Han, Yongjun Zhu, Shanxiong Chen, Lihua Peng, Yueqiang Xiao, Houhua Wu, Zhen Qu

**Affiliations:** 1 Department of Orthopedics, The Ninth People's Hospital of Chongqing, Chongqing, China; 2 College of Computer and Information Science, Southwest University, Chongqing, China; 3 Department of Orthopedics, Bishan Hospital of Chongqing Medical University (Bishan Hospital of Chongqing), Chongqing, China; 4 Department of Orthopedics, Zizhong County People's Hospital, Neijiang, Sichuan, China

**Keywords:** artificial intelligence, computer-assisted diagnosis, convolutional neural network, deep learning, femoral-neck fractures, garden classification, hip imaging, radiographs

In the published article, a Funding source was erroneously omitted from the Funding statement. The funder Chongqing Medical Scientific Research Project (Joint Project of Chongqing Health Commission and Science and Technology Bureau), grant 2025MSXM117, awarded to Xueyang Han, was not included.

The corrected Funding statement is:

The author(s) declared that financial support was received for the research and/or publication of this article. This research was funded by the Innovation and Development Joint Fund of Chongqing Natural Science Foundation (Grant No. CSTB2023NSCQ-LZX0013), the Sci-Tech Think Tank Research Project of Chongqing Association for Science and Technology (Grant No. 2024KXKT10), and the Chongqing Medical Scientific Research Project (Joint Project of Chongqing Health Commission and Science and Technology Bureau) (Grant No. 2025MSXM117) awarded to Xueyang Han. The funders provided financial support for the research design, data collection, analysis, and manuscript preparation. The APC was not funded by any specific grant.

The original version of this article has been updated.

