# Supplementary material for: Development and validation of a deep learning-based automatic detection and classification model for femoral neck fractures using hip imaging: a retrospective multicenter diagnostic study
Source: Front Med (Lausanne). 2026 Apr 8;13:1803858. doi: 10.3389/fmed.2026.1803858 (PMC13099755; doi:10.3389/fmed.2026.1803858)
Supplement: Supplementary file 1 [file Data_Sheet_1.pdf]

# Supplementary Material

## 1 SUPPLEMENTARY TABLES AND FIGURES

### 1.1 Tables

**Table S1.** Patient's clinical characteristics and basic information

| Characteristics | Development data set (n = 423) | Independent test data set (n = 106) | External validation data set (n = 277) |
|---|---|---|---|
| Age (years) | $69.5 \pm 9.5$ | $73.2 \pm 11.2$ | $72.0 \pm 10.3$ |
| Sex | Female: 220 (52%) | Female: 76 (71.7%) | Female: 154 (55.6%) |
| | Male: 203 (48%) | Male: 30 (28.3%) | Male: 123 (44.4%) |
| Hereditary | Yes: 89 (21%) | Yes: 30 (28%) | Yes: 83 (30%) |
| | No: 334 (79%) | No: 76 (72%) | No: 194 (70%) |
| Condition of Injury | Mobility: 207 (49%) | Mobility: 46 (43%) | Mobility: 110 (40%) |
| | Cane/stair: 216 (51%) | Cane/stair: 60 (57%) | Cane/stair: 167 (60%) |
| Living Conditions | Smoking: 254 (60%) | Smoking: 59 (56%) | Smoking: 172 (62%) |
| | Drinking: 287 (68%) | Drinking: 69 (65%) | Drinking: 194 (70%) |
| Medical History | Falls: 296 (70%) | Falls: 72 (68%) | Falls: 199 (72%) |
| | Fractures: 106 (25%) | Fractures: 31 (29%) | Fractures: 50 (18%) |
| | No Relevant History: 21 (5%) | No Relevant History: 3 (5%) | No Relevant History: 28 (10%) |
| Cause of Injury | Traffic Accident: 25 (6%) | Traffic Accident: 9 (8%) | Traffic Accident: 27 (10%) |
| | Fall: 304 (72%) | Fall: 74 (70%) | Fall: 224 (81%) |
| | High-Energy Injuries: 94 (22%) | High-Energy Injuries: 23 (22%) | High-Energy Injuries: 26 (9%) |

**Table S2.** Patient data statistics

| Data Type | Development data set (k = 6255) | Independent test data set (k = 1563) | External validation data set (k = 2192) |
|---|---|---|---|
| CT | 2128 (66%) | 422 (27%) | 921 (42%) |
| X-Ray | 4127 (34%) | 1141 (73%) | 1271 (58%) |

**Table S3.** Distribution of patients by age profile

| Age range | Development data set (n = 423) | Independent test data set (n = 106) | External validation data set (n = 277) |
|---|---|---|---|
| 0-10 | 0 (0%) | 0 (0%) | 0 (0%) |
| 10-20 | 4 (1%) | 3 (3%) | 3 (1%) |
| 20-30 | 12 (3%) | 4 (4%) | 0 (0%) |
| 30-40 | 21 (5%) | 8 (8%) | 0 (0%) |
| 40-50 | 33 (8%) | 8 (8%) | 22 (8%) |
| 50-60 | 46 (11%) | 14 (13%) | 33 (12%) |
| 60-70 | 105 (25%) | 20 (19%) | 72 (26%) |
| 70-80 | 126 (29%) | 26 (25%) | 77 (28%) |
| 80-90 | 67 (16%) | 14 (13%) | 50 (18%) |
| >90 | 9 (2%) | 9 (7%) | 20 (7%) |

**Table S4.** Comparison of Accuracy of Different Qualified Physician Groups and Models

| Groups | Garden I | Garden II | Garden III | Garden IV |
|---|---|---|---|---|
| Expert Group | 0.8235 (0.7662, 0.8694) | 0.8018 (0.7421, 0.8536) | 0.8979 (0.8515, 0.9331) | 0.9107 (0.8703, 0.9428) |
| Senior Group | 0.6614 (0.5960, 0.7215) | 0.6439 (0.5774, 0.7056) | 0.7576 (0.7013, 0.8092) | 0.8027 (0.7486, 0.8485) |
| Novice Group | 0.3847 (0.3220, 0.4481) | 0.4032 (0.3406, 0.4650) | 0.4746 (0.4112, 0.5389) | 0.5193 (0.4531, 0.5825) |
| Our Model | 0.9789 (0.9712, 0.9863) | 0.9594 (0.9490, 0.9692) | 0.9205 (0.9064, 0.9339) | 0.9454 (0.9339, 0.9568) |

## 1.2 Figures

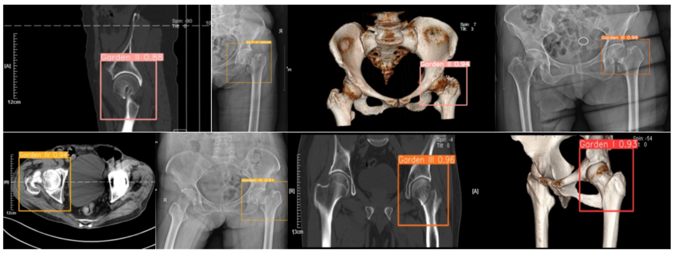

**Figure S1.** Visualization of hip femoral neck fracture detection and classification
